# Diagnostic value of cardiovascular magnetic resonance in comparison to endomyocardial biopsy in cardiac amyloidosis: a multi-centre study

**DOI:** 10.1007/s00392-020-01771-1

**Published:** 2020-11-10

**Authors:** Grigorios Chatzantonis, Michael Bietenbeck, Ahmed Elsanhoury, Carsten Tschöpe, Burkert Pieske, Gloria Tauscher, Julia Vietheer, Zornitsa Shomanova, Heiko Mahrholdt, Andreas Rolf, Sebastian Kelle, Ali Yilmaz

**Affiliations:** 1 Department of Cardiology I, University Hospital Münster, Albert-Schweitzer-Campus 1, Building A1, 48149 Münster, Germany; 2 Department of Cardiology, Centre for Regenerative Therapies (BCRT), Campus Virchow and Berlin Institute of Health (BIH), Berlin, Charite, Berlin, Germany; 3 DZHK (German Centre for Cardiovascular Research), partner site Berlin, Charite, Berlin, Germany; 4 Department of Internal Medicine/Cardiology, Deutsches Herzzentrum Berlin, Berlin, Germany; 5 Department of Cardiology, Robert-Bosch-Medical Centre, Stuttgart, Germany; 6 Department of Cardiology, Kerckhoff Hospital, University Giessen, Bad Nauheim, Germany; 7 DZHK (German Centre for Cardiovascular Research), partner site Rhine Main, Frankfurt, Germany

**Keywords:** CMR, CA, EMB, Scintigraphy, Immunofixation

## Abstract

**Background:**

Cardiac amyloidosis (CA) is an infiltrative disease characterised by accumulation of amyloid deposits in the extracellular space of the myocardium—comprising transthyretin (ATTR) and light chain (AL) amyloidosis as the most frequent subtypes. Histopathological proof of amyloid deposits by endomyocardial biopsy (EMB) is the gold standard for diagnosis of CA. Cardiovascular magnetic resonance (CMR) allows non-invasive workup of suspected CA. We conducted a multi-centre study to assess the diagnostic value of CMR in comparison to EMB for the diagnosis of CA.

**Methods:**

We studied *N* = 160 patients characterised by symptoms of heart failure and presence of left ventricular (LV) hypertrophy of unknown origin who presented to specialised cardiomyopathy centres in Germany and underwent further diagnostic workup by both CMR and EMB. If CA was diagnosed, additional subtyping based on EMB specimens and monoclonal protein studies in serum was performed. The CMR protocol comprised cine- and late-gadolinium-enhancement (LGE)-imaging as well as native and post-contrast T1-mapping (in a subgroup)—allowing to measure extracellular volume fraction (ECV) of the myocardium.

**Results:**

An EMB-based diagnosis of CA was made in *N* = 120 patients (CA group) whereas *N* = 40 patients demonstrated other diagnoses (CONTROL group). In the CA group, *N* = 114 (95%) patients showed a characteristic pattern of LGE indicative of CA. In the CONTROL group, only 1/40 (2%) patient showed a “false-positive” LGE pattern suggestive of CA. In the CA group, there was no patient with elevated T1-/ECV-values without a characteristic pattern of LGE indicative of CA. LGE-CMR showed a sensitivity of 95% and a specificity of 98% for the diagnosis of CA. The combination of a characteristic LGE pattern indicating CA with unremarkable monoclonal protein studies resulted in the diagnosis of ATTR-CA (confirmed by EMB) with a specificity of 98% [95%-confidence interval (CI) 92–100%] and a positive predictive value (PPV) of 99% (95%-CI 92–100%), respectively. The EMB-associated risk of complications was 3.13% in this study—without any detrimental or persistent complications.

**Conclusion:**

Non-invasive CMR shows an excellent diagnostic accuracy and yield regarding CA. When combined with monoclonal protein studies, CMR can differentiate ATTR from AL with high accuracy and predictive value. However, invasive EMB remains a safe invasive gold-standard and allows to differentiate CA from other cardiomyopathies that can also cause LV hypertrophy.

**Electronic supplementary material:**

The online version of this article (10.1007/s00392-020-01771-1) contains supplementary material, which is available to authorized users.

## Introduction

Cardiac amyloidosis (CA) is caused by deposition of misfolded amyloid fibrils in the extracellular space of the myocardium resulting in a specific cardiomyopathy [1, 2]. The two major forms of CA are transthyretin-related amyloidosis (ATTR) and immunoglobulin light-chain amyloidosis (AL), accounting for nearly 95% of cases [3]. ATTR comprises two subtypes: the acquired wild-type (wt) ATTR (wtATTR) and the hereditary or mutant ATTR (mATTR) that is caused by genetic mutations in the transthyretin (TTR) gene [1, 4].

Cardiovascular magnetic resonance (CMR) has emerged as a robust non-invasive imaging modality that offers comprehensive and detailed cardiac information regarding functional and structural data [5]. In particular, late-gadolinium-enhancement (LGE)-imaging allows to robustly detect a characteristic pattern of myocardial damage indicative of CA [6]. Moreover, novel CMR techniques such as T1-mapping and measurement of the extracellular volume fraction (ECV) of the myocardium promise an improved assessment of even subtle structural changes in the myocardium. Some recent single-centre studies suggested a higher diagnostic yield and prognostic value of CMR based on T1-mapping and ECV measurement compared to conventional LGE-imaging [7, 8]. However, multi-centre CMR data regarding the diagnostic yield of CMR parameters for the diagnosis of CA are still limited.

Despite considerable progress in non-invasive imaging modalities, invasive endomyocardial biopsy (EMB) is still considered the gold standard for workup of cardiomyopathies of unknown origin [9–11]. The need for invasive EMB was recently questioned by Gillmore et al. in case of suspected CA since the combined finding of a “positive” bone scintigraphy indicative of CA and the absence of monoclonal proteins resulted in a specificity as well as positive predictive value (PPV) of 100% for the diagnosis of cardiac ATTR [12].

In the present multi-centre study, we sought to assess the diagnostic value of CMR regarding the diagnosis of CA in patients with unexplained left ventricular hypertrophy (LVH)—in comparison to the current gold-standard EMB. Similar to previous scintigraphic studies [12], we analysed the specificity and PPV of CMR in patients with the combined finding of a “positive” CMR study indicative of CA and “negative” monoclonal protein studies.

## Methods

### Study population

**Fig. 1:**
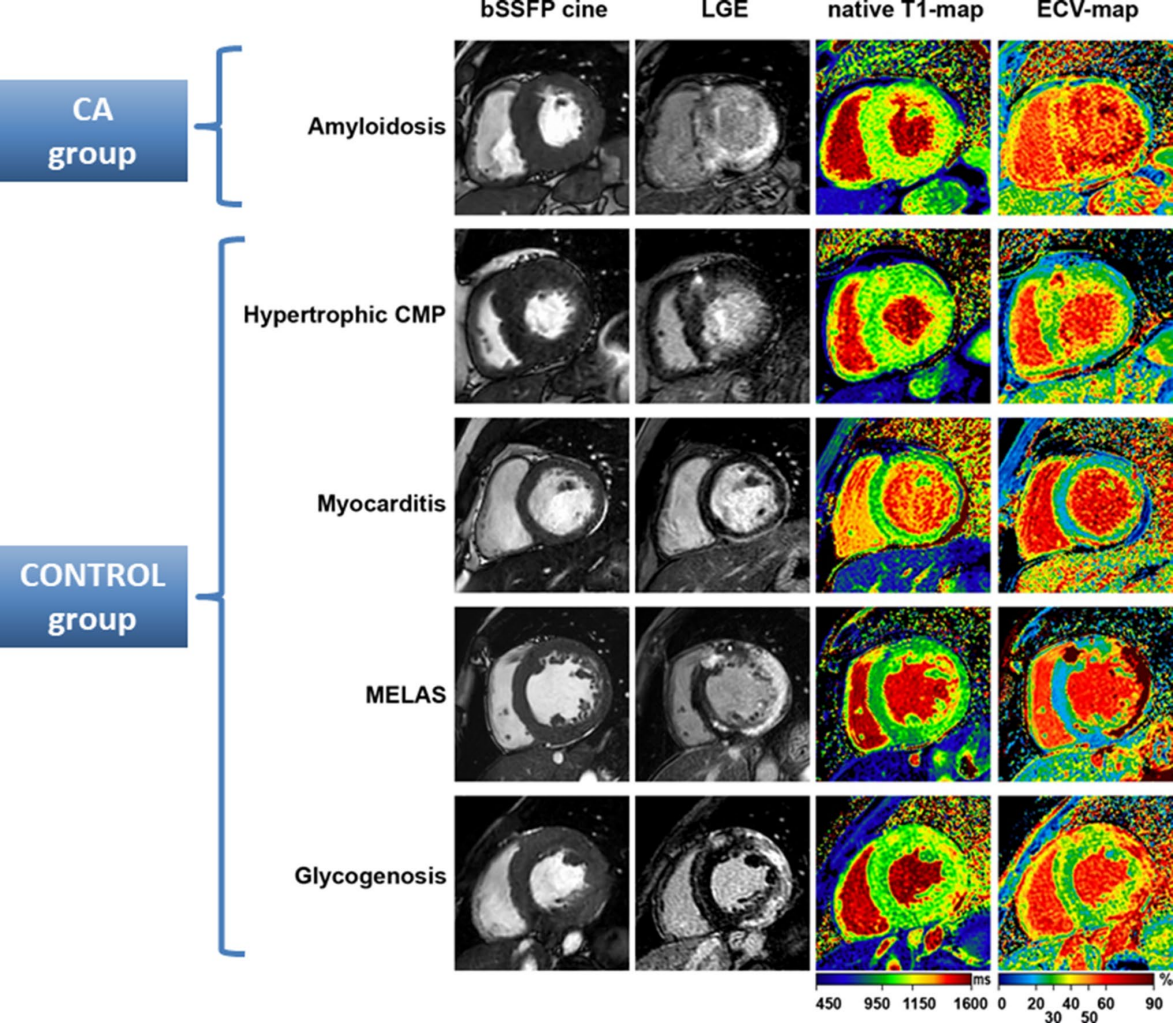
Cardiovascular magnetic resonance (CMR) images of a patient with a characteristic pattern of late-gadolinium-enhancement (LGE) indicative of cardiac amyloidosis (CA)—in comparison to findings in the control group. Apart from LGE images, native T1-maps and ECV-maps are shown

The present study group comprised *N* = 160 patients suffering from symptoms of heart failure in the presence of left ventricular (LV) hypertrophy (defined as maximal LV wall thickness of ≥12 mm) that could not be explained by abnormal loading conditions and who were referred to four German specialised cardiomyopathy centres between 2016 and 2019 for further diagnostic workup. Only patients who underwent both non-invasive CMR and invasive EMB, respectively, were included in this retrospective study. Noteworthy, patients with e.g. typical CMR findings of hypertensive heart disease or hypertrophic cardiomyopathy (HCM) who did not routinely undergo EMB were not included in this analysis. Moreover, the participating German centres share similar diagnostic algorithms including EMB workup in every patient with suspected CA. If EMB workup resulted in the diagnosis of CA, additional subtyping based on EMB-specimens was performed to differentiate ATTR from AL (and other forms). Furthermore, if CA was diagnosed based on CMR and/or EMB, additional monoclonal protein studies—comprising serum protein electrophoresis (sPE), serum immunofixation electrophoresis (sIFE) and serum-free light chain assay (sFLC)—were performed. In case of an EMB-based diagnosis of ATTR, additional genetic testing of the transthyretin gene was ordered to differentiate between wild-type (wt)-ATTR and mutant (m)-ATTR. Patients with an EMB-based diagnosis of CA (either ATTR or AL) were assigned to the CA group whereas patients with other cardiac diagnoses formed the CONTROL group (Fig. 1). Ethics approval was obtained by local authorities and written informed consent was obtained by participants.

### CMR acquisition

CMR studies were performed on 1.5-T scanners (either Ingenia or Achieva, Philips Healthcare, Best, the Netherlands or Magnetom Aera, Siemens Medical Systems, Erlangen, Germany) in *N* = 151 patients and on a 3.0-T scanner (Skyra, Siemens Medical Systems, Erlangen, Germany) in *N* = 9 patients. The CMR protocol comprised cine- and LGE-imaging [magnitude only and additional phase-sensitive inversion recovery (PSIR) in case of need] as well as native and post-contrast T1-mapping using a modified Look Locker inversion recovery (MOLLI) sequence (performed in 3 out of 4 participating centres and *N* = 93 out of 160 patients)—allowing to measure extracellular volume fraction (ECV) of the myocardium. A CMR-based diagnosis of CA was made if a characteristic LGE-pattern indicative of CA was present. T1-mapping images were obtained in three short-axis views prior to as well as 15–20 min after intravenous contrast agent administration (Gadobutrol or Gd-DOTA 0.15 mmol/kg) to determine native T1- and ECV-values. Patients with chronic kidney disease (CKD) of stage 4 or 5 [i.e. glomerular filtration rate (GFR) ≤30 ml/min] were not excluded by default. Furthermore, patients with cardiac devices were only excluded if they showed fractured, epicardial or abandoned leads prior to the CMR study and/or poor image quality due to large device artefacts during the CMR scan.

### CMR data analysis

**Fig. 2:**
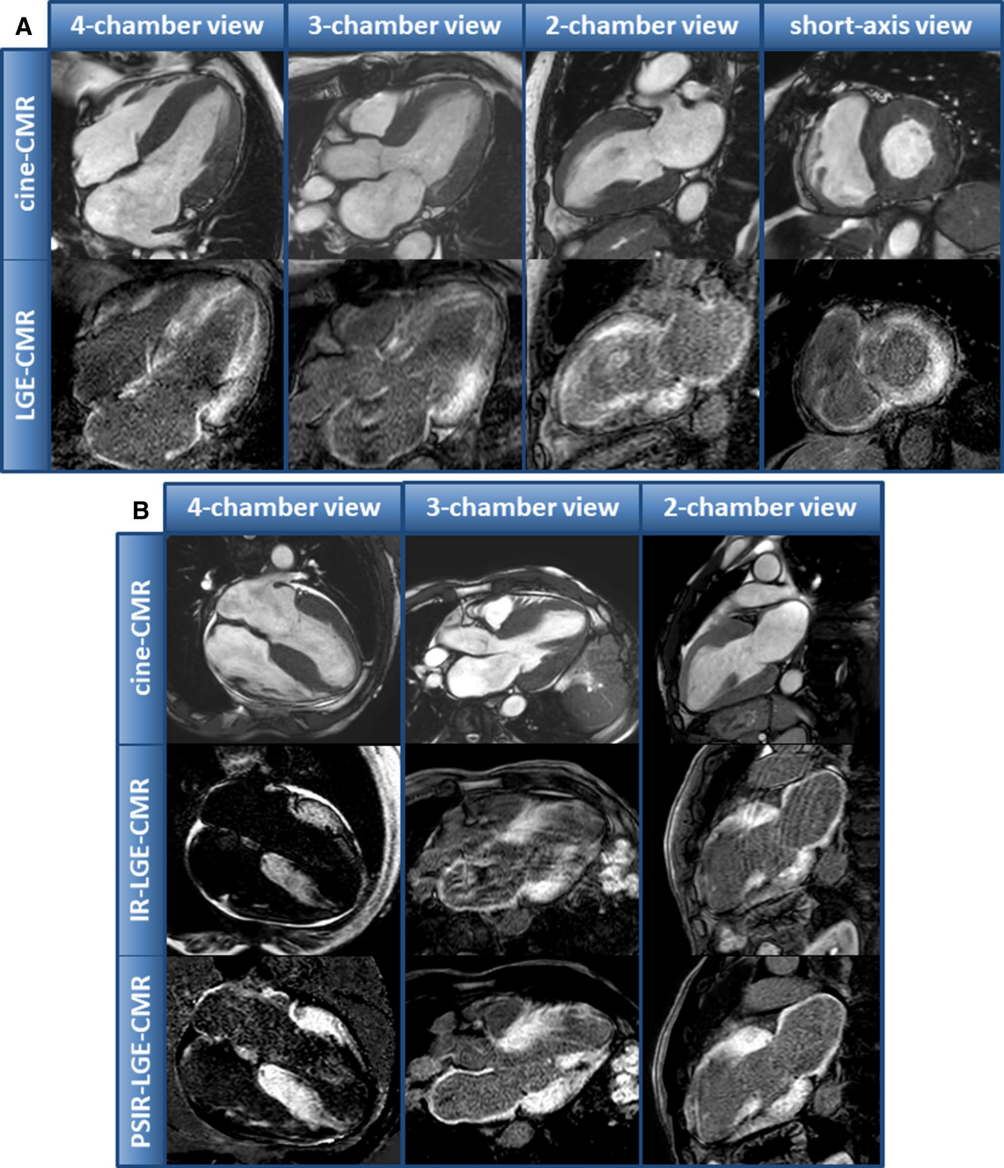
**a** Example of a patient with ATTR amyloidosis and a characteristic late-gadolinium-enhancement (LGE)-pattern indicative of cardiac amyloidosis. **b** Example of another patient with ATTR amyloidosis and both magnitude only and additional phase-contrast inversion recovery (PSIR) LGE-images. PSIR-LGE-images show an improved image contrast and allow a better delineation of LGE in this example

CMR image analysis and interpretation were performed using commercially available software (cvi42—version 5.11.0, Circle Cardiovascular Imaging, Calgary, Alberta, Canada). Analysis of ventricular volumes and function as well as LV mass was made by contouring the endocardium and epicardium in short-axis cine-images. First, LGE-images were assessed visually and the pattern, distribution and extent of LGE was used to derive a CMR-based diagnosis of the underlying cardiac disease—as illustrated in detail elsewhere [18]. A CMR-based diagnosis of CA was supposed if a characteristic LGE-pattern indicative of CA (comprising all of the following criteria) was observed: (1) subendocardial to transmural LGE pattern predominantly in the basal LV segments, (2) no LGE distribution correlating to the perfusion area of a coronary artery and suggesting an ischemic myocardial scar, (3) no sharp demarcation and rather diffuse and extensive LGE pattern (Fig. 2a). In case of poor image quality of magnitude-only LGE images, additional PSIR-LGE were assessed (Fig. 2b). LGE was described as non-characteristic, when the aforementioned criteria were only partially fulfilled. In addition, the “Query Amyloid Late Enhancement” (QALE) score was reported as described in more detail elsewhere [13]. T1- and ECV-maps were assessed based on the consensus statement of SCMR. In those *N* = 9 patients who were studied at 3.0-T, only ECV values (but not absolute T1-values) were considered for further analyses. All CMR data analyses were performed offline by experienced readers.

### Monoclonal protein studies

Patients with suspected CA were tested for the presence of monoclonal gammopathy by studying the presence of monoclonal proteins in serum—suggestive of AL. First, sPE with high-resolution agarose gel allowed the demarcation of an M-gradient. If an M-protein was present, sIFE and sFLC were performed to characterise its immunoglobulin chain type (heavy vs. light). The presence of a monoclonal protein was defined as an abnormal sFLC ratio (kappa-lambda ratio <0.26 or >1.65) or presence of a monoclonal band in sIFE.

### EMB procedure

EMB specimens were obtained either from the RV septal wall (via a femoral vein access site using a 7F long-sheath with an angulated tip) and/or from the LV free wall (via a femoral artery access site using a 7F long-sheath without angulation following a retrograde approach) [14, 15] (Supplemental Figure 1). Continuous ECG, blood pressure and pulse oximetry monitoring were performed throughout the whole procedure. Fluoroscopy was used for guidance of the long-sheath and bioptome, and for targeting the region of interest—which was defined in advance by non-invasive CMR. At least four EMB samples were collected from the RV and/or LV. Post-procedural echocardiography was performed to rule out or detect a pericardial effusion possibly caused by the biopsy procedure. Definition and assessment of biopsy complications was in accordance with previous studies [11].

### EMB workup (histology and immunohistochemistry)

Histopathological and molecular pathological workup of biopsy samples were performed as described in detail previously [11, 16]. Myocardial inflammation indicative of myocarditis was defined on the basis of immunohistochemical analyses based on hematoxylin/eosin and Masson trichrome stainings, respectively. Furthermore, Masson trichrome and hematoxylin/eosin staining as well as electron microscopy allowed EMB-based diagnoses of (amongst others) hypertrophic cardiomyopathy (HCM), dilated cardiomyopathy (DCM), cardiac sarcoidosis, transplant rejection, LV non-compaction cardiomyopathy (LVNC), mitochondrial cardiomyopathy, toxic cardiomyopathy, glycogen-storage disease and CA, respectively. Detection of amyloid was initially performed with Congo red staining of the formalin-fixed, paraffin-embedded myocardial tissue samples. Subsequently, immunohistochemistry allowed to identify the type of protein subunit with the use of monospecific antibodies reactive with the various types of amyloid. A negative result for CA was presumed only after thorough investigation by a specialised pathologist confirming negative histological and immunohistochemical examinations. Additional electron microscopy studies were performed depending on the preceding clinical and imaging suspicion and the pathologist’s individual assessment.

### Genetic testing

In those patients with EMB-proven ATTR amyloidosis, additional testing regarding the presence of a TTR gene mutation was in general aimed at. However, due to medicolegal reasons, genetic testing was only performed in *N* = 56 out of 160 patients. Informed written consent for genetic testing in accordance with the Genetic Diagnostics Act (GenDG) was obtained. Thereafter, DNA extraction from the patient’s blood with amplification by polymerase chain reaction assay and sequencing of the coding region of the TTR gene was performed. Mutations in the TTR gene associated with amyloid deposition are described elsewhere.

### Statistical analysis

Statistical analysis was performed with SPSS (version 26.0, IBM Corp., Armonk, NY). Normal distribution of numeric variables was assessed with Shapiro–Wilk test. Continuous variables are expressed as mean ± standard deviation whereas skewed variables as median ± interquartile range. Categorical variables are expressed as frequency with percentage. An independent two-sample *T* test was used for comparison of normally distributed data and Mann–Whitney *U* test for non-normally distributed variables. The Fisher's exact test was used to compare non-continuous variables. Receiver operating characteristic curves (ROC) were analysed to assess the specificity and sensitivity of different CMR measurements to identify patients with CA within the whole study group. A *p* value <0.05 was considered statistically significant. Sensitivity and specificity of CMR in diagnosing CA as well as positive (PPV) and negative predictive values (NPV) were calculated accordingly. Confidence intervals (CI) for sensitivity and specificity were calculated with the exact Clopper–Pearson method; CI for the predictive values was given as standard logit CI.

## Results

### Study population

The characteristics of the study population are summarised in Table 1. An EMB-based diagnosis of CA was made in *N* = 120 patients (CA group) whereas *N* = 40 patients demonstrated other diagnoses (CONTROL group). Males and females were equally distributed in the CA and CONTROL group (*p* = 0.48). Median age differed significantly between the two groups [75 (68–80) years in CA vs. 52 (46–61) years in CONTROLs; *p* < 0.001] as expected due to the higher prevalence of CA in elderly patients. Atrial fibrillation was more common in CA patients (49 vs. 25%; *p* = 0.010).

**Table 1:** Patient characteristics

	CA group *N* = 120	CONTROL group *N* = 40	*P* value
Male, *n* (%)	100 (83)	31 (78)	0.48
Age, years	75 (68–80)	52 (46–61)	** <0.001**
BMI, kg/m^2^	26 (±4)	26 (±6)	0.87
Hypertension, *n* (%)	87 (73)	22 (55)	0.05
Diabetes, *n* (%)	21 (18)	4 (10)	0.32
High cholesterol, *n* (%)	64 (53)	11 (28)	**0.006**
Current smoker, *n* (%)	15 (13)	15 (38)	**0.001**
Coronary artery disease, *n* (%)	48 (40)	8 (20)	**0.023**
Atrial fibrillation, *n* (%)	59 (49)	10 (25)	**0.010**
Chronic kidney disease (GFR < 30 ml/min), *n* (%)	11 (9)	0 (0)	0.07

Bold *P* values indicate *P* < 0.05

### Endomyocardial biopsy (EMB) findings

EMB was performed in all patients of the study group within a narrow time window after the CMR study [median time from CMR to EMB = 1 (0–3) months]. Regarding EMB-associated complications, there were four patients with myocardial perforation resulting in pericardial effusion with need for pericardiocentesis and one patient with a long-sheath induced non-sustained ventricular tachycardia. Importantly, all complications were treated quickly and successfully, and there were no detrimental or persistent complications at all. The overall complication rate of the EMB procedure was 3.13%.

The diagnosis of CA in EMB samples was made according to the aforementioned histopathological definitions in *N* = 120 patients. Amyloid-subtyping by targeted immunohistochemistry resulted in the diagnosis of cardiac ATTR amyloidosis in *N* = 92 (77%) patients and cardiac AL amyloidosis in *N* = 28 (23%) patients. In the remaining *N* = 40 CONTROL patients without cardiac amyloidosis, however, with the presence of LV hypertrophy per definition, the following diagnoses were obtained: *N* = 10 HCM, *N* = 9 myocarditis, *N* = 3 cardiac sarcoidosis, *N* = 3 transplant rejection, *N* = 3 LVNC, *N* = 3 DCM, *N* = 2 mitochondrial cardiomyopathy, *N* = 2 toxic cardiomyopathy, *N* = 1 glycogen-storage disease, *N* = 1 cardiac involvement in filaminopathy and *N* = 3 non-ischemic cardiomyopathy of unknown aetiology.

### Genetic transthyretin testing results

The majority of those *N* = 92 patients with biopsy-proven ATTR amyloidosis underwent additional TTR gene testing for potential pathogenic TTR gene mutations. Mutations in the TTR gene indicating the presence of mATTR were detected in *N* = 6 patients. The remaining patients with biopsy-proven ATTR (94%) were classified as wtATTR.

### CMR findings

The detailed anatomic, functional and structural CMR findings are listed in Table 2. Left ventricular ejection fraction (LV-EF) was significantly reduced in the CONTROL group compared to the CA group [40 (32–51) % vs. 57 (46–62) %; *p* < 0.001]. Accordingly, LV end diastolic volume (LV-EDV) was markedly increased in the CONTROL group compared to the CA group (116 ± 41 ml/m^2^ vs. 77 ± 22 ml/m^2^; *p* < 0.001). Due to our study inclusion criteria, LV hypertrophy was present in all study patients—without a relevant difference in (global) LV mass between the CA and the CONTROL group (*p* = 0.69). However, a significantly higher maximal LV wall thickness was observed in the CA group compared to CONTROLs [19 [17–22] mm vs. 14 (13–18) mm; *p* < 0.001].

**Table 2:** CMR parameters

	CA group *N* = 120	CONTROL group *N* = 40	*P* value
Conventional CMR parameters			
LV-EF, %	57 (46–62)	40 (32–51)	** <0.001**
LV-EDV index, ml/m^2^	77 (±22)	116 (± 41)	** <0.001**
LV-ESV index, ml/m^2^	31 (23–42)	61 (44–99)	** <0.001**
LV mass index, g/m^2^	91 (71–105)	90 (68–110)	0.69
LV mass/volume ratio, g/ml	1.17 (0.98–1.34)	0.83 (0.65–1.07)	** <0.001**
Max. LV wall thickness, mm	19 (17–22)	14 (13–18)	** <0.001**
RV-EF, %	49 (±11)	51 (± 10)	0.41
RV-EDV index, ml/m^2^	82 (±22)	92 (± 28)	0.06
RV-ESV index, ml/m^2^	39 (30–49)	44 (28–58)	0.34
LGE			
LGE extent, %	82 (52–100)	35 (20–59)	** <0.001**
LGE QALE score, *n*	12 (7–17)	4 (2–5)	** <0.001**
Characteristic LGE indicative of CA, *n* (%)	114 (95)	1 (2)	** <0.001**
T1-mapping			
Native T1-mapping global, ms	1152 (1095–1245)	1043 (1036–1104)	** <0.001**
Native T1-mapping basal septal, ms	1139 (1086–1233)	1062 (1018–1137)	**0.001**
ECV			
ECV global, %	52 (±10)	34 (±10)	** <0.001**
ECV basal septal, %	53 (43–63)	31 (27–39)	** <0.001**

Bold *P* values indicate *P* < 0.05

Regarding myocardial structure analyses, a non-ischemic, diffuse subendocardial to transmural pattern of LGE predominantly present in the LV basal to midventricular segments was detected in CA patients—with a substantially higher myocardial LGE extent in comparison to the CONTROL group [82 (52–100) % vs. 35 (20–59) %, *p* < 0.001]. Accordingly, the assessment of the QALE score resulted in a significantly increased score in the CA group compared to the CONTROL group [12 (7–17) vs. 4 (2–5); *p* < 0.001]. Furthermore, both native T1- and ECV-values were significantly increased in the CA group compared to the CONTROLs—not only in the basal septal wall but also in case of global LV assessment. In the CA group, there was no patient with elevated T1-/ECV-values without a characteristic pattern of LGE indicating CA (in those patients with available T1-maps). Noteworthy, neither native T1- nor ECV-values were available in those six patients with EMB-based diagnosis of CA, but absence of a LGE-pattern indicative of CA.

**Fig. 3:**
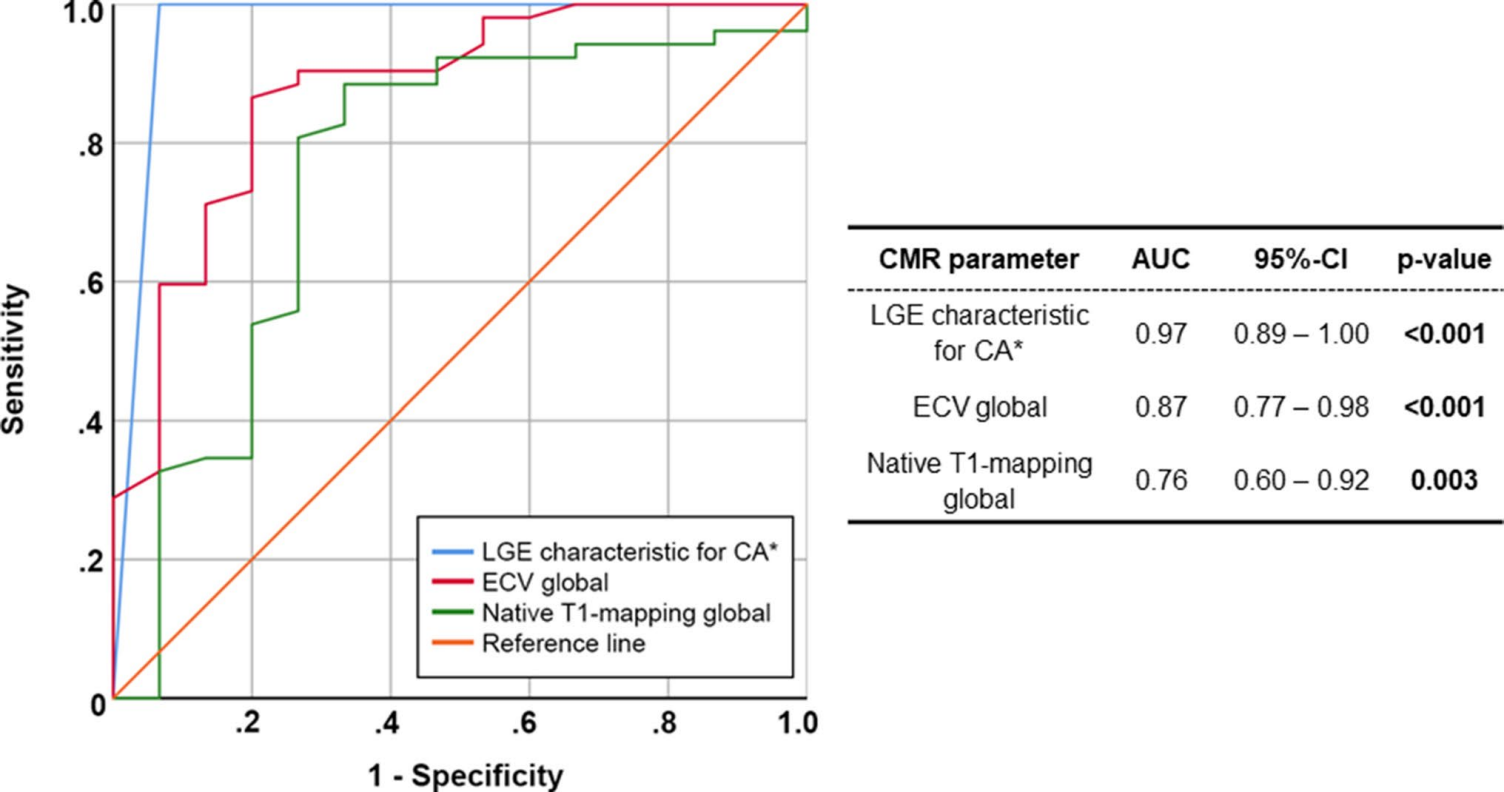
Receiver operating characteristic (ROC) curves illustrating the diagnostic yield of different CMR parameters regarding the diagnosis of cardiac amyloidosis (CA)

Subsequent ROC analyses showed excellent diagnostic yield for the parameters (a) predefined characteristic pattern of LGE indicative of CA, (b) global ECV—and a slightly reduced diagnostic value for (c) global native T1-mapping regarding the delineation of CA patients from the CONTROL group (Fig. 3). In particular, the parameter “characteristic pattern of LGE indicative of CA” (assessed as characteristic for CA according to the aforementioned criteria) showed an area under the curve (AUC) of 0.97 (95%-CI: 0.89–1.00; *p* < 0.001) whereas global ECV had an AUC of 0.87 (95%-CI: 0.77–0.98; *p* < 0.001) and global native T1 an AUC of 0.76 (95%-CI: 0.60–0.92; *p* = 0.003).

LGE-imaging findings were further validated in comparison to biopsy findings. The respective LGE findings were classified dichotomously into “characteristic LGE pattern indicative of CA” vs. “LGE pattern NOT unequivocally indicative of CA”. There was only one patient with a “characteristic LGE pattern indicative of CA” that could not be verified by EMB. Review of this patient’s case showed that a RV biopsy was performed and that the bioptome was not optimally positioned in the basal to mid part of the interventricular septum but rather in the apical part. Hence, a potential “sampling error” due to suboptimal positioning of the bioptome may have resulted in a “false-negative” EMB result. Furthermore, there were 6 out of 120 (5%) patients with EMB-proven CA who did not show a “characteristic LGE pattern indicative of CA” upon CMR. In comparison to the gold standard EMB, the non-invasive CMR parameter “characteristic LGE pattern indicative of CA” showed a sensitivity of 95% and a specificity of 98% for the diagnosis of CA (Table 3). Accordingly, the positive predictive value (PPV) of LGE-CMR for the diagnosis of CA was 99% (95%-CI: 94–100%) whereas the respective negative predictive value (NPV) was 87% (95%-CI: 75–93%).

**Table 3:** Sensitivity and specificity of CMR for the diagnosis of CA compared to EMB

	Characteristic LGE indicative of CA	LGE pattern NOT indicative of CA	Sensitivity and specificity (CI), %
EMB-proven presence of CA, *N* = 120	114 (95)	6 (5)	95 (89–98) sensitive
EMB negative for CA, *N* = 40	1 (2)	39 (98)	98 (87–100) specific

### Combination of CMR and monoclonal protein findings

Presence of monoclonal proteins in the serum suggestive of AL was found in all *N* = 28 patients with biopsy-proven AL amyloidosis. In contrast, there were 6 out of 92 (7%) patients with biopsy-proven ATTR amyloidosis who also showed presence of serum monoclonal proteins—in the absence of AL amyloidosis. Hence, monoclonal protein findings in these six patients with a median age of 73 years were assessed as monoclonal gammopathy of undetermined significance (MGUS). The combined finding of a “characteristic LGE pattern indicative of CA” (upon CMR) AND negative monoclonal protein studies was 98% specific for the diagnosis of cardiac ATTR amyloidosis. Moreover, such a combined finding showed a PPV of 99% (95%-CI 92–100%) for the diagnosis of cardiac ATTR amyloidosis when compared to biopsy findings (Table 4).

**Table 4:** Combination of CMR results with monoclonal protein studies (MPS)

	Characteristic LGE indicative of CA + negative MPS	LGE pattern NOT indicative of CA and/or positive MPS	Sensitivity and specificity (CI), %	PPV and NPV (CI), %
EMB-proven presence of ATTR-CA, *N* = 92	83	9	90 (82–95) sensitive	NPV = 88 (80–93)
EMB negative for ATTR-CA, *N* = 68	1	67	99 (92–100) specific	PPV = 99 (92–100)

## Discussion

To the best of our knowledge, this is the largest multi-centre German study that evaluated the diagnostic value of CMR in comparison to EMB-proven CA. The results of our present study are based on real-world clinical data from four German centres that are highly experienced in both (a) conducting CMR studies (with a high volume of >2.000 CMR studies per year and centre) and (b) performing EMBs (with a high volume of >100 EMB procedures per year and centre) for workup of suspected cardiomyopathies of unknown origin. This real-world German experience clearly illustrates that (a) non-invasive CMR allows to diagnose the presence of CA with a high sensitivity of 95% and an even higher specificity of 98% and that (b) the combined finding of a positive CMR study (indicative of CA) and negative monoclonal protein studies is not only highly specific for the diagnosis of cardiac ATTR amyloidosis (specificity of 98%) but also highly trustable with a PPV of 99% (for the diagnosis of cardiac ATTR amyloidosis)—proven by biopsy results.

Already in 2008, the Stuttgart group assessed the diagnostic value of LGE-CMR for the diagnosis of CA in a rather small study group of 33 patients [6]: cardiac amyloidosis was detected by EMB in 15 out of 33 patients who were studied more than a decade ago (on 1.5-T Magnetom Sonata at that time). Using the characteristic LGE-pattern indicative of CA as a diagnostic criterion, they obtained a sensitivity of 80% and a specificity of 94% for the diagnosis of CA—at that time. Obviously, both clinical experience in assessing LGE-CMR images as well as CMR-techniques have tremendously improved since that time allowing (amongst others) more experienced and more accurate assessment of LGE-images today [17–19]. Hence, the results of the present multi-centre study nicely illustrate this overall progress and clearly demonstrate that both sensitivity and specificity of LGE-based diagnosis of CA have improved.

Obviously, today cardiac workup of patients with suspected CA should be based on multi-parametric CMR including T1-mapping and ECV measurement—and not limited to LGE-CMR—since several studies have shown a superior diagnostic value of mapping-based approaches compared to conventional LGE-imaging [7, 8]. In principle, native T1-mapping and ECV measurement are highly suitable tools to detect (and quantify) even subtle amyloid deposits in the extracellular space of the myocardium [5, 20]. However, there are still some puzzling data regarding the comparison of native T1- and ECV-values in case of CA, e.g. the “native T1 versus ECV paradox” in CA that was recently addressed in detail elsewhere [21]. In a recent meta-analysis, a total of 18 diagnostic (*N* = 2015) and 13 prognostic CMR studies (*N* = 1483) using native T1, ECV or LGE to diagnose and prognosticate CA were included for analysis [22]. According to this meta-analysis, the parameter ECV showed a significantly higher diagnostic odds ratio for CA than conventional LGE-assessment. However, there was no significant difference between LGE-assessment and native T1 for sensitivity, specificity and diagnostic odds ratio—regarding the diagnosis of CA. Our present study does not allow to safely compare the diagnostic value of T1-mapping and/or ECV measurement in comparison to LGE-imaging since mapping was not performed in all patients. In this context, it needs to be considered that cut-off values for native T1 or ECV derived from ROC analyses (in a specific group of study patients) for ruling in or out the presence of CA are—amongst others—determined by native T1- and ECV-values of the respective control group. A different composition of the control group (e.g. higher percentage of HCM patients with extensive myocardial fibrosis vs. lower percentage of healthy controls without fibrosis) will result in different cut-off values. Hence, comparisons of diagnostic CMR parameters based on ROC analyses (using cut-off values) need to be considered carefully—with a special attention to the control group of the underlying study—and single-centre results cannot be transferred to other centres by default.

In the last years, innumerable studies addressing non-invasive diagnosis of CA were published by colleagues from the National Amyloidosis Centre in London/UK—deserving the designation the London/UK experience [7, 12, 17, 23]. Their study results moved the diagnostic field forward and substantially contributed to current recommendations regarding the diagnostic approach in suspected CA [12, 24, 25]. Due to the efforts of these colleagues, bone scintigraphy was established as a substantial non-invasive method to diagnose CA [24–27]. However, this London/UK experience needs to be considered with some caution and a non-reflected transfer of the UK-based results to other EU countries should be avoided. As outlined by these colleagues themselves, the patients presenting to this central UK centre are not “unselected”, but rather referred to this centre of experience with suspected CA. Consequently, the cohort of patients studied at this centre—and in particular, the “control group” used in their studies—does not reflect an “unselected” cardiology population of patients. Obviously, the same is true for our present study due to our methodological approach in selecting patients. Importantly, the patients of this study were initially referred as “outpatients” to our specialised cardiomyopathy centres (mostly by resident cardiologists due to suspected cardiomyopathy for further diagnostic workup). Hence, we neither included, e.g. decompensated inpatients presenting directly to our emergency or intensive care units who also underwent CMR and/or biopsy workup during their further hospitalisation, nor did we study an unselected cardiology population. However, our control group comprising only patients with LV hypertrophy should be more appropriate regarding validation of imaging methods for the diagnosis of CA.

To appropriately assess the value of the present CMR results in comparison to previous bone scintigraphy results (e.g. the London/UK experience), a careful look on the data of Gillmore et al. is required [12]: in a first step, Gillmore et al. analysed the data of 374 patients with EMB and defined a “positive” bone scintigraphy indicative of CA as cardiac tracer uptake of either grade 1, 2 or 3 (vs. grade 0 as “normal” finding). This approach resulted in a sensitivity of 88% and a specificity of 87% for bone scintigraphy to detect CA (independent of subtype). In a second step, the authors focused on those patients with ATTR-CA and received a very high sensitivity of >99% for bone scintigraphy to detect ATTR-CA—but a rather low specificity of 68%. In a third step, the authors changed their “positive” definition of bone scintigraphy and presumed that a cardiac tracer uptake of only grade 2 or 3 was indicative of ATTR-CA (vs. grade 0 or 1 defined as “normal/negative” findings). Such a different assumption resulted in a re-grouping of 42 (out of 374 patients = 11%) from the positive group to the negative group, and in a sensitivity of 91% and a specificity of 87% for bone scintigraphy to detect ATTR-CA. In comparison, in our present CMR study, the presence of CA was diagnosed based on LGE-CMR with a high sensitivity of 95% and an even higher specificity of 98%—without re-grouping of study patients by changing assumptions.

**Fig. 4:**
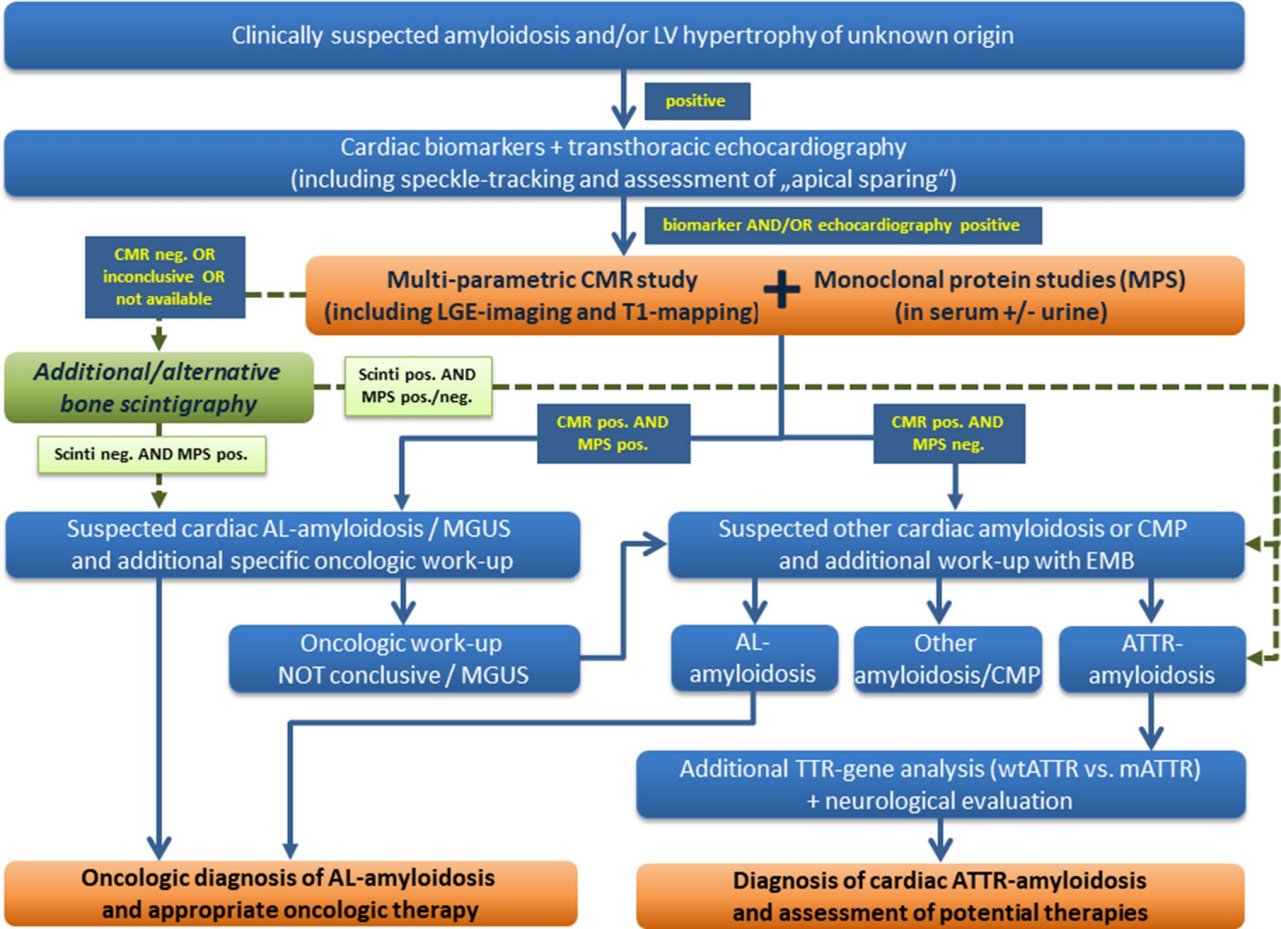
Schematic diagram representing the suggested diagnostic pathway for workup of cardiac amyloidosis (CA)

Furthermore, Gillmore et al. analysed the specificity and PPV of a “positive” bone scintigraphy indicative of CA (defined as cardiac tracer uptake of either grade 2 or 3 vs. grade 0 or 1 as “normal/negative”) for ATTR-CA when combined with the absence of monoclonal proteins in *N* = 374 patients with EMB [12]: they obtained a specificity as well as PPV of 100% for the diagnosis of ATTR-CA. Based on this finding, they established their approach and diagnostic algorithm of bone scintigraphy-based non-biopsy diagnosis of ATTR-CA that entered some recommendation papers [24–26]—and is questioned by some interesting reports [28, 29]. In contrast, in our present study, the combined finding of a “positive” CMR study indicative of CA and “negative” monoclonal protein studies resulted in a similar specificity of 98% and PPV of 99% for the diagnosis of ATTR-CA (compared to biopsy findings). Hence, our present results clearly suggest that non-invasive CMR allows both (a) to safely diagnose the presence of CA and (b) to further verify the presence of ATTR-CA by additional negative monoclonal protein studies—similar to bone scintigraphy. Obviously, in case of such clear CMR findings there is no need for additional diagnostic methods (such as bone scintigraphy)—and the diagnostic algorithm suggested in some recommendation papers needs to be carefully revised. Based on the present German experience, we suggest the diagnostic algorithm illustrated in Fig. 4.

Finally, one may argue that additional EMBs are not required in those patients with the combined finding of a “positive” CMR study indicative of CA and “negative” monoclonal protein studies—similar to the scintigraphic approach of Gillmore et al. [12]. However, since our knowledge on the underlying pathophysiology of amyloid deposition in the human myocardium is still very limited, and since CA is not only characterised by extracellular accumulation of amyloid fibrils but also by myocardial oedema, inflammation, and myocyte hypertrophy (with variable degrees of each pathophysiology potentially resulting in differing effects on native T1, ECV and LGE-pattern), we still need the histopathological information from EMBs in order to better understand our non-invasive imaging findings. This issue will be even far more important considering upcoming specific therapies to treat ATTR-CA: we will need the EMB information in order to better understand both (a) the therapeutic effect of the respective medication/therapy and (b) the change in non-invasive cardiac imaging parameters that will be used for disease monitoring. Hence, considering the low risk of EMB complication even in patients with CA—as confirmed in the present study, we suggest to continue to biopsy even patients with the combined finding of a “positive” CMR study indicative of CA and “negative” monoclonal protein studies. For the remaining patients, there is no doubt that EMB still represents the gold standard for workup of non-ischemic, unexplained cardiomyopathy [9, 10, 16].

Obviously, the EMB complication rate of 3.13% in the present study is somewhat higher compared to previous studies suggesting a major complication rate of 0.64% for LV-EMB and of 0.82% for RV-EMB—proving that EMB is a safe procedure. Moreover, there are reports that mentioned a myocardial perforation risk (= major complication) in CA patients in up to 17.1% in case of RV-EMB and up to 6.6% in case of LV-EMB (Kristen et al. Am J Hematology 2007;82:327–333)—and suggested a more “fragile” myocardium in case of CA with a higher risk of myocardial perforation in particular in case of RV-EMB. Obviously, the complication rate of EMB in the present study was tremendously lower as reported in the aforementioned study of Kristen et al. In the present study, myocardial perforation occurred in four patients all belonging to the CA group—supporting the notion of a “fragile” myocardium in case of advanced CA. However, these complications occurred when a “stiff” bioptome was used whereas no complications were observed when using more smooth/elastic bioptomes. In our opinion, the risk of perforation in case of EMB is driven by (a) the properties of the bioptome, (b) the experience of the interventionalist and (c) the composition of the myocardium—with a potentially more “fragile” myocardium in case of CA. Therefore, EMB procedures in patients with suspected CA are quite safe when they are performed by experienced interventionalists and preferably taken from the LV using smooth bioptomes.

Finally, the issue of balancing diagnostic yield vs. procedure-associated risk and costs is highly important regarding clinical decision-making in daily routine. However, a discussion on this issue would go far beyond the scope of the present work and should be performed by expert panels based on more comprehensive data regarding all of these issues. Nevertheless, we believe that the “diagnostic” data presented in our manuscript will help such expert panels aiming at balancing diagnostic yield, procedure safety, method availability, costs and practical consequences.

## Limitations

Similar to other previous studies, our study group does not reflect an “unselected” cardiology population of patients and comprised only those suffering from symptoms of heart failure in the presence of left ventricular (LV) hypertrophy that could not be explained by abnormal loading conditions. Obviously, we cannot exclude a potential selection bias by selected patient referral to our specialised cardiomyopathy centres. However, we believe that our approach is appropriate for evaluating the diagnostic yield of CMR for the workup of CA (based on real-world clinical data), since our CONTROL group did not comprise healthy patients or patients without relevant structural abnormalities—but rather severely diseased patients with other cardiac diseases presenting to four German specialised cardiomyopathy centres. Moreover, T1-mapping and ECV data were not available in all patients—limiting the value of comparative analyses of LGE vs. native T1 and/or ECV. However, LGE-pattern per se showed a convincing diagnostic yield and T1-mapping-based approaches will obviously “strengthen”—but not “worsen”—this yield and particularly allow a monitoring of myocardial amyloid load over time.

## Conclusion

Non-invasive CMR shows an excellent diagnostic accuracy and yield regarding CA. When combined with monoclonal protein studies, CMR can differentiate ATTR from AL with high accuracy and predictive value. However, invasive EMB remains a safe invasive gold standard and allows to differentiate CA from other cardiomyopathies that can also cause LV hypertrophy.

## Electronic supplementary material

Below is the link to the electronic supplementary material.

Supplementary file1 (TIF 298 kb)

## Data Availability

The datasets used and/or analysed during the current study are available from the corresponding author on reasonable request.

## Abbreviations

AL: Immunoglobulin light chain amyloidosis
ATTR: Transthyretin amyloidosis
CA: Cardiac amyloidosis
CAD: Coronary artery disease
CIED: Cardiovascular implantable electronic device
CKD: Chronic kidney disease
CMR: Cardiovascular magnetic resonance
ECG: Electrocardiogram
ECV: Extracellular volume
EMB: Endomyocardial biopsy
HCM: Hypertrophic cardiomyopathy
HFpEF: Heart failure with preserved ejection fraction
IQR: Interquartile range
LGE: Late-gadolinium enhancement
LV: Left ventricle
LV-EDV: Left ventricular end-diastolic volume
LV-EF: Left ventricular ejection fraction
LVH: Left ventricular hypertrophy
MGUS: Monoclonal gammopathy of undetermined significance
NPV: Negative predictive value
NSF: Nephrogenic systemic fibrosis
PPV: Positive predictive value
QALE: Query amyloid late enhancement
ROC: Receiver operating characteristic curve
ROI: Region of interest
sFLC: Serum-free light chain assay
sIFE: Immunofixation electrophoresis in serum
SPE: Serum protein electrophoresis
TTE: Transthoracic echocardiogram
