# Supplementary figures and images for: Diagnostic value of cardiovascular magnetic resonance in comparison to endomyocardial biopsy in cardiac amyloidosis: a multi-centre study
Source: Clin Res Cardiol. 2020 Nov 10;110(4):555–68. doi: 10.1007/s00392-020-01771-1 (PMC8055632; doi:10.1007/s00392-020-01771-1)

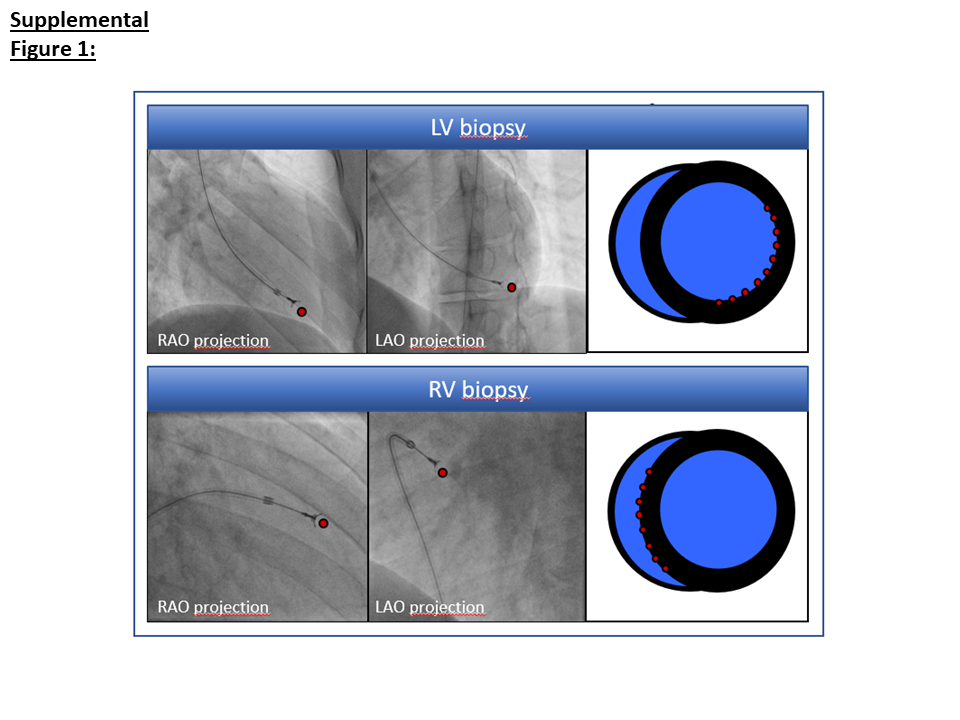

Supplement: Supplementary file 1 — Supplementary file1 (TIF 298 kb) [file 392_2020_1771_MOESM1_ESM.tif]
